# Hypothermia induced alteration of repolarization - impact on acute and long-term outcome: a prospective cohort study

**DOI:** 10.1186/s13049-017-0417-6

**Published:** 2017-07-11

**Authors:** Sophie von Ulmenstein, Christian Storm, Thomas G. K. Breuer, Sebastian Lask, Philipp Attanasio, Andreas Mügge, Alexander Wutzler

**Affiliations:** 1 Department of Cardiology, Charité - Universitaetsmedizin Berlin, Campus Virchow-Klinikum, Augustenburger Platz 1, 13353 Berlin, Germany; 2 Department of Nephrology and Intensive Care Medicine, Charité - Universitaetsmedizin Berlin, Campus Virchow-Klinikum, Berlin, Germany; 3 Department of Internal Medicine of the Ruhr-University Bochum, St. Josef-Hospital, Bochum, Germany; 4 Heart and Vascular Center of the Ruhr-University Bochum, St. Josef-Hospital, Bochum, Germany

## Abstract

**Background:**

The effects of target temperature management (TTM) on the heart aren’t thoroughly studied yet. Several studies showed the prolongation of various ECG parameters including Tpeak-Tend-time under TTM. Our study’s goal is to evaluate the acute and long-term outcome of these prolongations.

**Methods:**

In this study we included patients with successful resuscitation after cardiac arrest who were admitted to the Charité Virchow Klinikum Berlin or the Heart and Vascular Centre of the Ruhr University Bochum between February 2006 and July 2013 (Berlin) or May 2014 to November 2015 (Bochum). For analysis, one ECG during TTM was recorded after reaching the target temperature (33–34 °C) or in the first 6 h of TTM. If possible, another ECG was taken after TTM. The patients were being followed until February 2016.

Primary endpoint was ventricular arrhythmia during TTM, secondary endpoints were death and hospitalization due to cardiovascular diseases during follow-up.

**Results:**

One hundred fifty-eight patients were successfully resuscitated in the study period of which 95 patients had usable data (e.g. ECGs without artifacts). During TTM significant changes for different parameters of ventricular de- and repolarization were noted: QRS (103.2 ± 23.7 vs. 95.3 ± 18.1; *p* = 0.003),QT (405.8 ± 76.4 vs. 373.8 ± 75.0; *p* = 0.01), QTc (474.9 ± 59.7 vs. 431.0 ± 56.8; *p* < 0.001), JT (302.8 ± 69.4 vs. 278.5 ± 75.2; *p* = 0.043), JTc (354.3 ± 60.2 vs. 318.7 ± 59.1; *p* = 0.001). 13.7% of the patients had ventricular arrhythmias during TTM, however these patients showed no difference regarding their ECG parameters in comparison to those were no ventricular arrhythmias occurred. We were able to follow 69 Patients over an average period of 35 ± 31 months. The 14 (21.5%) patients who died during the follow-up had significant prolongations of the TpTe-time in the ECGs without TTM (103.9 ± 47.2 vs. 75.8 ± 28.6; *p* = 0.023).

**Conclusion:**

Our results show a significant prolongation of ventricular repolarization during TH. However, there was no significant difference between the ECG parameters of those who developed a ventricular arrhythmia and those who did not. The temporary prolongation of the repolarization during TTM seems to be less important for the prognosis of the patient. Whereas the prolongation of the repolarization in the basal ECG is associated with a higher mortality in our study.

## Background

The European Resuscitation Council included target temperature management (TTM) in its guidelines as a method to prevent patients after cardiac arrest from neurological damage [1, 2]. Nonetheless, its effect on the heart is still not certain [3].

In the last years, several studies examined the effect of TTM on different ECG parameters. The results suggest that TTM causes the prolongation of various intervals such as the QT interval and the Tpeak-to-Tend (TpTe) interval but without an increase in life-threatening arrhythmias [4, 5].

The TpTe interval reflects the transmural dispersion of repolarization and is acknowledged to be a helpful parameter to evaluate the risk of severe arrhythmic events [6, 7]. However, its impact on patients under TTM and its role as a prognostic marker for the acute and long-term outcome of these patients is not thoroughly studied.

The purpose of this study is to evaluate if it is possible to establish TpTe and its relation to the QT interval as a risk marker for cardiac arrest in patients who had undergone TTM.

## Methods

### Participants

In this prospective cohort study patients with cardiac arrest and successful resuscitation (survival over 24 h) were included. Due to current guidelines and our local standard protocol all survivors after cardiac arrest received TTM if they remained comatose at time of admission, despite location or type of cardiac arrest.

All the patients were admitted to the Charité Virchow Klinikum Berlin or the Heart and Vascular Centre of the Ruhr University Bochum between February 2006 and July 2013 (Berlin) or May 2014 to November 2015 (Bochum) and underwent TTM at a target temperature of 33 to 34 °C.

### ECG analysis

We analyzed one ECG under hypothermia at 33.0 °C, which was taken after reaching the target temperature or in the first 6 h of TTM, and if available another ECG without hypothermia at least 24 h after TTM. The manual measurements of most of the intervals were obtained from lead V1 or from the next readable chest lead. Only the PQ interval was measured in lead II.

TpTe was defined as the interval of the highest or lowest point of the T wave to the point where the T wave intersects the isoelectric line. For biphasic T waves the highest or lowest part of the first wave was taken and measured until the end of the second wave. TpTe was put into relation with the QT interval. The QT interval was corrected with Bazett’s Formula.

QRS fragmentation was characterized as QRS >120 ms without a bundle branch block (BBB), notched R/S in two consecutive leads, an RSR-configuration but QRS <120 ms and no incomplete RBBB, >1 R’ in two consecutive leads or QRS >120 ms and >2 R’.

The QT dispersion in this study was obtained from the longest and the shortest QT interval in a 12-lead-ECG.

### Follow up

The Follow up of the patients was performed until February 2016. New hospitalizations were registered and their charts were reviewed. In addition, the German population register was used to follow up for the death of patients who were not admitted once more to the Charité or the Ruhr University Bochum.

### Data analysis

For all statistical analyses SPSS 21 (IBM, Armonk, NY, USA) was utilized. To compare the ECG parameters with and without hypothermia a paired t-test and Wilcoxon’s test was conducted. Furthermore, a logistic regression and a Kaplan-Meier survival-analysis were performed.

Primary endpoint was ventricular arrhythmia during TTM, secondary endpoints were death and hospitalization due to cardiovascular diseases during follow-up.

## Results

### Study population

In our study, 158 patients were included between February 2006 and November 2015. After reevaluation of the ECGs 63 patients had to be excluded due to no hypothermia, no readable ECG, no available chest leads or indeterminable ECG date. Unfortunately, 39 patients did not have an ECG without hypothermia in their patient files and thus could only be used for the Baseline characteristics and the long-term outcome.

The average age was 59.1 ± 16.2 and the patients were mainly male (80%). In most cases the initial rhythm was ventricular fibrillation (VF; 65.6%) and the cardiac arrest was caused by an acute myocardial infarction (AMI; 52.6%). During hospitalization 26 patients died. The mean ascertained APACHE II Score at admission was 27.6 ± 8.3. Typical concomitant diseases were coronary artery disease (CAD; 67.4%), hypertension (46.3%), heart failure (33.7%) and renal failure (25.3%). All baseline characteristics are shown in Table 1.

**Table 1:** Baseline and follow-up characteristics

Baseline characteristics	Patients (*n* = 95)
Age	59.1 (± 16.2)
Male sex	76 (80%)
Initial rhythm	
VF	63 (65.6%)
Asystole	25 (26%)
VT	3 (3.1%)
PEA	5 (5.2%)
Lay resuscitation	36 (37.5%)
Myocardial infarction	50 (52.6%)
Cardiomyopathy	6 (6.3%)
LVEF (%)	40.3 (± 13.8)
Died during hospitalization	26 (27.1%)
APACHE II	27.6 (± 8.3)
Time on ventilation (h)	309.8 (± 273.2)
Time on ICU (d)	19.1 (± 15.0)
CAD	64 (67.4%)
Previous myocardial infarction	9 (9.5%)
Previous ICD/Pacemaker	4 (4.2%)
Diastolic dysfunction	2 (2.1%)
Heart failure	32 (33.7%)
COPD	8 (8.4%)
Hypertension	44 (46.3%)
Renal failure	24 (25.3%)
Follow-up characteristics	Patients
Death	27.8%
Newly diagnosed ventricular arrhythmia	5.6%
ICD shock	3.7%
Newly diagnosed SVT	7.4%
AMI	1.9%

*VF ventricular fibrillation, VT ventricular tachycardia, PEA pulseless electrical activity, LVEF left ventricular ejection fraction, ICU intensive care unit, CAD coronary artery disease, COPD chronic obstructive pulmonary disease, SVT supraventricular tachycardia, AMI acute myocardial infarction*

Values are mean (SD) or numbers (%)

A good outcome at discharge according to the Cerebral Performance Category scale (CPC 1 or 2) was seen in 58.9% of the patients.

A follow-up was performed until February 2016. Sixty nine patients were followed during an average period of 35 ± 31 months. Fifteen patients were lost to follow-up. 21 of the 54 remaining follow-up patients did not have another hospitalization at the Charité and hence, we could only collect the information if they died during our observation period. Moreover, three patients had ventricular arrhythmia, two patients had an ICD shock, one patient had another AMI and 15 patients died (Table 1).

### ECG analysis

ECGs were taken during hypothermia and afterwards. Several ECG parameters increased significantly during hypothermia (Table 2): QRS (103.2 ± 23.7 vs. 95.3 ± 18.1; *p* = 0.003), QT (405.8 ± 76.4 vs. 373.8 ± 75.0; *p* = 0.01), QTc (474.9 ± 59.7 vs. 431.0 ± 56.8; *p* = 0.000), JT (302.8 ± 69.4 vs. 278.5 ± 75.2; *p* = 0.043), JTc (354.3 ± 60.2 vs. 318.7 ± 59.1; *p* = 0.001). Although there was an increase in the duration of these parameters, especially QT/QTc, only 11.6% of the patients developed ventricular tachycardia and 2.1% ventricular fibrillation during hypothermia. Furthermore, the QT/QTc interval and TpTe were not significantly longer in patients who developed these arrhythmias (QT: *p* = 0.158; QTc: *p* = 0.138; TpTe: *p* = 0.297; Table 3).

**Table 2:** Difference of ECG parameters during and after TTM

Measurements	TTM	After TTM	n	*p*-Value
PQ (ms)	159.0 (± 34.0)	147.3 (± 33.2)	40	.059
QRS (ms)	103.2 (± 23.7)	95.3 (± 18.1)	56	.002
QT (ms)	405.8 (± 76.4)	373.8 (± 75.0)	56	.010
QTc (ms)	474.9 (± 59.7)	431.0 (± 56.8)	56	.000
QTd (ms)	101.0 (± 46.2)	93.0 (± 46.8)	56	.234
JT (ms)	302.8 (± 69.4)	278.5 (± 75.2)	56	.043
JTc (ms)	354.3 (± 60.2)	318.7 (± 59.1)	56	.002
TpTe (ms)	92.4 (± 44.2)	80.5 (± 33.3)	54	.051
TpTe-QT-Ratio	0.23 (± 0.08)	0.21 (± 0.07)	54	.240
TpTe d	67.9 (± 39.9)	56.1 (± 35.1)	56	.099
Heart rate (−min)	86.3 (± 20.5)	85.2 (± 25.9)	56	.806

*TTM targeted temperature management*

**Table 3:** Ventricular arrhythmia during TTM

	No ventricular arrhythmia	Ventricular arrhythmia	*p*-Value
PQ (ms)	163.5 (± 31.0)	171.3 (± 29.4)	0.437
QRS (ms)	102.9 (± 24.3)	96.9 (± 29.8)	0.610
QT (ms)	398.4 (± 75.0)	365.8 (± 86.8)	0.158
QTc (ms)	463.8 (± 61.4)	435.0 (± 81.9)	0.138
QTd (ms)	95.8 (± 44.9)	104.2 (± 44.4)	0.530
JT (ms)	295.4 (± 67.8)	268.9 (± 75.1)	0.200
JTc (ms)	343.9 (± 60.3)	319.2 (± 69.9)	0.183
TpTe (ms)	92.9 (± 38.8)	85.4 (± 28.5)	0.297
TpTe-QT-Ratio	0.23 (± 0.07)	0.24 (± 0.10)	0.643
TpTe d	67.7 (± 37.0)	68.5 (± 60.1)	0.380
Heart rate (−min)	85.5 (± 21.1)	89.3 (± 20.7)	0.547

*TTM targeted temperature management, TpTe Tpeak-Tend-Time*

Values are means (standard deviation)

There was no significant difference in the heart rate (86.3 ± 20.5 vs. 85.2 ± 25.9; *p* = 0.806). The PQ interval could not be measured for several ECGs due to atrial fibrillation, junctional rhythm or paced rhythm.

### Long-term ECG results

Sixty nine patients were followed over a period of 35 months ±31 months. Fourteen patients died during this period (21.5%). Patients who died during follow-up showed a significant longer TpTe in the ECGs after TTM (103.9 ± 47.2 vs. 75.8 ± 28.6; *p* = 0.023 Table 4) in comparison to long-term survivors, while QT and QTc values were not significantly different between the groups.

There was no significant difference in the 180-days-survival between the groups of normal or prolonged interval for the parameters QTc, TpTe and TpTe-QT-ratio during TTM and after TTM.

**Table 4:** Death after discharge

	No incidence	Death	*p*-Value
PQ (ms)	151.0 (± 32.9)	161.6 (± 60.5)	0.718
QRS (ms)	94.1 (± 19.3)	98.9 (± 17.6)	0.476
QT (ms)	374.0 (± 59.5)	410.0 (± 124.5)	0.420
QTc (ms)	422.9 (± 46.7)	464.9 (± 86.2)	0.191
QTd (ms)	91.9 (± 45.7)	90.0 (± 53.4)	0.697
JT (ms)	279.9 (± 60.3)	311.1 (± 124.9)	0.484
JTc (ms)	315.15 (± 48.7)	346.9 (± 92.4)	0.344
TpTe (ms)	75.8 (± 28.6)	103.9 (± 47.2)	0.023
TpTe-QT-Ratio	0.20 (± 0.07)	0.25 (± 0.05)	0.076
TpTe d	59.0 (± 37.9)	48.9 (± 17.6)	0.667
Heart rate(−min)	80.33 (± 21.0)	88.2 (± 38.6)	0.567

*TTM targeted temperature management, TpTe Tpeak-Tend-time*

ECG after TTM. Values are means (standard deviation)

## Discussion

This study is to our knowledge the first one to analyze the effect of TTM on different ECG parameters and the long-term outcome in a large study population. Hypothermia can lead to a prolongation of ventricular repolarization, which subsequently can cause ventricular arrhythmias [6, 8].

The mechanisms behind the prolongation of PR and QT intervals during hypothermia are complex and are partly due to serum electrolyte changes, which include hypokalemia, increased intracellular Calcium and Sodium concentration [9, 10]. The Calcium overload is most likely the main factor behind severe cardiac arrhythmias [9, 10]. Moreover the myocardium doesn’t cool down homogenously which leads to repolarization differences in the tissue and thus can also cause arrhythmias [10].

Piktel et al. showed that TTM at 32 °C slightly increased the TpTe in canines, but with a decreased arrhythmogenesis and with TpTe going back to baseline values after rewarming in comparison to canines which underwent severe hypothermia at 26 °C [11].

As the study from Piktel et al. was conducted in healthy canines without previous resuscitation, the results are most likely to vary from the effects of TTM in resuscitated patients. Our results are consistent with previous studies regarding the acute effect of TH in showing a prolongation of QT/QTc – with no significant prolongation of QTd and TpTe - and without an increase of severe arrhythmic events [4, 5]. Moreover, there was no significant difference regarding the ECG parameters in patients with ventricular arrhythmia during TTM and patients without ventricular arrhythmias. Hence, the temporary prolongation of the ECG parameters doesn’t seem to be of prognostic importance. Although the TpTe interval was not significantly prolonged during TTM, patients who died after their hospitalization had a significant longer TpTe interval in general. This is consistent with previous studies that evaluated the TpTe as a marker of sudden cardiac death in the general population [12].

### Limitations

No ECGs were available in advance to the hypothermia. However, another study showed that the QT time is decreasing after hypothermia to baseline levels [4]. Hence, it can be assumed that the QT time after hypothermia is the same as before. Moreover our study was conducted in patients with a target temperature at 33–34 °C. The new guidelines however allow a temperature between 32 and 36 °C, thus the effects on the heart could vary from our results in a different temperature management [13].

Additionally, the study was performed without a control group of patients, who didn’t receive TTM after cardiac arrest. Therefore, the ECG changes during TTM could also be caused by other factors, e.g. ventricular adaptation after cardiac arrest. It might be useful to conduct a similar study with a control group to exclude such a phenomenon. However, since TTM is standard treatment in postresuscitation care, it is difficult to define a control group. The best match would be a group of patients after cardiac arrest that was not treated with TTM, e. g. a historical control group. Unfortunately, this was not possible at our hospitals and therefore, a control group could not be added.

## Conclusion

Our study showed a significant prolongation of the ventricular repolarization under TTM. However, this prolongation doesn’t correlate with the incidence of ventricular arrhythmias. Therefore, it’s not advisable to use it as a prognostic marker.

Nonetheless, it might be worth thinking about establishing TpTe as a risk marker in ECGs without TTM. Further studies about this ECG parameter might be useful.
